# Short- and long-term effectiveness of physical activity interventions for women living with and beyond breast cancer: a systematic review and meta-analysis

**DOI:** 10.1007/s12282-026-01859-y

**Published:** 2026-04-27

**Authors:** Alejandro Dominguez Garcia, Barbara A. Mullan, Kylie Hill, Lauren J. Breen, Chloe Maxwell-Smith

**Affiliations:** 1 Behavioural Science and Health Research Group, Curtin University, Kent Street, Bentley, WA 6102 Australia; 2 School of Population Health, Curtin University, Bentley, WA Australia; 3 Curtin enAble Institute, Curtin University, Bentley, WA Australia; 4 Curtin School of Allied Health, Curtin University, Bentley, WA Australia; 5 Curtin Medical Research Institute, Cancer Domain, Curtin University, Bentley, WA Australia

**Keywords:** Breast cancer, Exercise, Physical activity, Systematic review, Meta-analysis

## Abstract

**Background:**

Physical activity is beneficial for women living with and beyond breast cancer. Many interventions aim to increase physical activity in this population; however, their effectiveness is uncertain. This review examines changes in physical activity levels across short- and long-term timepoints following these interventions.

**Methods:**

Online databases (CINAHL Ultimate, Medline, ProQuest, PsycINFO, SCOPUS, and Web of Science) were systematically searched for studies evaluating physical activity interventions for women living with and beyond breast cancer, assessing physical activity outcomes at baseline, post-intervention (short-term), and at least 3 months from the end of the intervention (long-term). Random-effects multilevel models were used for meta-analyses.

**Results:**

The review included 43 studies with 5295 participants. Of these, 28 had sufficient data for meta-analyses. For between-group analyses, moderate-to-vigorous physical activity (MVPA) showed moderate effects at post-intervention (SMD = 0.46, 95% CI [0.17, 0.75]), while total physical activity showed small-to-moderate effects (SMD = 0.33, [0.15, 0.51]). At long-term follow-up (range 3 months to 5 years), effects were small for MVPA (SMD = 0.24, [0.14, 0.35]) and total physical activity (SMD = 0.25, [0.08, 0.43]). For within-group analyses, MVPA showed small-to-moderate effects (SMD = 0.38, [0.12, 0.63]) at post-intervention, while total physical activity showed moderate effects (SMD = 0.46, [0.14, 0.77]). At long-term follow-up, change from baseline MVPA was not statistically significant, while total physical activity showed a moderate effect (SMD = 0.33, [0.18, 0.47]).

**Conclusions:**

Physical activity interventions for women with and beyond breast cancer appear effective in the short term. However, long-term effects are inconsistent, especially for intensive activity. Identifying influential factors, including psychological determinants, that promote long-term physical activity would support sustained survivorship outcomes.

**Supplementary Information:**

The online version contains supplementary material available at 10.1007/s12282-026-01859-y.

## Introduction

Physical activity has numerous benefits during and beyond breast cancer treatment, including improving health-related quality of life and functioning, and reducing negative effects of treatment, cancer-related mortality, and recurrence rates [1, 2]. Individuals diagnosed with cancer are recommended to engage in at least 150 min of moderate or 75 min of vigorous aerobic exercise per week, in addition to two resistance training sessions (consistent with guidelines for the general population) [3–5]. However, most women living with and beyond breast cancer (80%) do not meet these recommendations [6]. Commonly reported physical activity barriers include nausea, cancer-related fatigue, and reduced physical functioning [6, 7]. Although interventions promoting physical activity in women living with and beyond breast cancer seem effective in the short term, the evidence for long-term change is inconsistent, necessitating investigation of whether short-term post-intervention changes are sustained [8, 9]. Given the benefits of physical activity, encouraging sufficient physical activity and maintaining exercise behaviours is crucial for optimising survivorship and health-related quality of life outcomes [10].

Systematic reviews have explored behavioural maintenance after physical activity interventions. However, some are not specific to breast cancer [11], limiting understandings about breast cancer-specific treatment trajectories, side effect profiles, and survivorship experiences [12]. Others have focused exclusively on exercise-based interventions, excluding those without a structured exercise component [13]. Most reviews analysed different subsets of studies when comparing short-term effects (baseline to post-intervention) and long-term effects (baseline to long-term follow-up) [11, 13, 14], limiting the comparability of effect trajectories. Pairwise meta-analysis models are typically used in reviews [9, 11, 13, 14]; however, unlike multilevel models, they do not adequately account for studies contributing multiple, non-independent effect sizes [15]. Therefore, this systematic review explored the behavioural effects of physical activity interventions for women living with and beyond breast cancer by evaluating changes in physical activity levels from (1) baseline to post-intervention, and (2) baseline to long-term follow-up (at least 12 weeks from end of intervention).

## Materials and methods

This review was conducted following the Preferred Reporting Items for Systematic Reviews and Meta-Analysis (PRISMA) guidelines [16] and pre-registered with the PROSPERO systematic review registry on 26 July 2024 (CRD42024573336). Any changes to the protocol are reported.

### Search strategy

The search for published articles was conducted in the following databases in September 2024: CINAHL Ultimate, Medline, ProQuest, PsycINFO, SCOPUS, and Web of Science. Search strategies were developed in collaboration with a health sciences librarian. Complete search terms and strategies are available in Supplemental Material 1.

### Eligibility criteria

Articles included for systematic review were (1) peer-reviewed and in English; (2) described an intervention targeting physical activity or exercise; (3) only involved women living with and beyond breast cancer or provided stratified data specific to women living with and beyond breast cancer; (4) did not involve other health behaviours (such as diet); (5) had a behavioural measure of physical activity levels, such as self-report questionnaires or accelerometers (not including measures of exercise capacity, such as VO_2_ max); and (6) had a long-term follow-up measure at least 12 weeks following the end of the intervention. For this review, “women” was defined as people assigned female at birth. Studies were not required to have a control group. For inclusion in the meta-analyses, studies had to provide means and standard deviations or sufficient data to estimate these (e.g., other measures of central tendency and variability), for physical activity levels at three time points: baseline, post-intervention, and long-term follow-up. In studies with more than one arm, only those arms which met these criteria were included. Studies that did not meet the criteria for the meta-analyses were described and summarised narratively. Where multiple articles reported on the same intervention, the most comprehensive or recent was used as the primary reference; however, relevant articles, including protocol papers, were consulted for additional study details as needed.

### Selection process

Search results were uploaded into EndNote (version 20.6, Clarivate) [17]. Duplicates and retracted articles were manually verified by the primary reviewer (ADG) and removed. Results were imported into Research Screener (https://researchscreener.com), a semi-automated machine-learning tool supporting systematic review abstract screening [18]. Duplicate titles and abstracts were identified and removed by Research Screener. Abstracts that were too long (> 30,000 characters), short (< 100 characters), or missing were manually reviewed by ADG. Records were removed if neither the abstract nor full article could be found or were confirmed as ineligible.

A screening tool (see Supplemental Material 2) based on the inclusion criteria was piloted by two independent reviewers over three rounds, with disagreements resolved by consensus. Abstracts were screened by ADG using Research Screener [18], which presents articles in ranked “blocks” of 50 based on relevance; screening ceased after two consecutive blocks contained no eligible articles.

After abstract screening, eligible articles were imported into Rayyan (https://www.rayyan.ai), a web-based systematic review manager. Additional duplicates were removed before full text-screening. Full-text screening was conducted by ADG and undergraduate research assistants using the refined inclusion criteria screening tool. The few disagreements were discussed and resolved by the reviewers. The reference lists of eligible articles, reviews, and meta-analyses identified throughout the screening process were checked by ADG for potential missed articles.

### Data extraction

Data were extracted independently by two reviewers (ADG and a research assistant). ADG extracted data from eligible studies with the assistance of SciSpace (https://scispace.com) [19], an AI-powered scientific reading and extraction tool for structured data retrieval from research articles. The research assistant extracted data manually. Disagreements were discussed and resolved by the reviewers. Extracted data included sample demographics, intervention descriptions, follow-up durations, and means and standard deviations for physical activity measures at all available time points. Corresponding authors of articles were contacted once via email if information was missing or unclear.

### Risk of bias assessment

Risk of methodological bias was assessed independently using the Joanna Briggs Institute (JBI) Critical Appraisal Tools for Randomised-Controlled Trials [20] and Quasi-experimental Studies [21]. The tools assess potential sources of bias with a checklist containing 13 questions for randomised controlled trials and nine for quasi-experimental studies. Reviewers answered each question with either ‘yes’, ‘no’, ‘unclear’, or ‘N/A’. Per updated guidelines, questions were addressed at the study, outcome, or result level, as appropriate [20, 21]. Study quality and risk of bias were summarised narratively [22].

### Statistical analysis

The initial protocol proposed that a conventional fixed- or random-effects meta-analytic model would be required. However, as several studies contributed multiple non-independent effect sizes, a multilevel random effects model was implemented in R (version 4.4.3) using the metafor package (version 4.8.0) [23] to account for clustering within studies [15].

A multilevel random-effects model accounted for the dependency structure of the data and expected heterogeneity across studies. For baseline-to-post analyses, a three-level model was specified (sampling variance; outcomes nested within studies; between-study variance). For baseline-to-follow-up analyses, a four-level model accounted for repeated measurements over time, adding repeated measures over time. Heterogeneity was assessed using *Q* (statistical significance of heterogeneity) and *I*^2^ (percentage of variance due to heterogeneity) statistics [15]. Egger’s tests assessed publication bias using the standard error of the effect sizes as a moderator in the multilevel model [24, 25]. Post hoc exploratory moderator analyses were not specified in our protocol. However, they were conducted when *I*^2^ exceeded 25% to examine whether study-level characteristics could explain any heterogeneity between effect sizes. Moderators tested included intervention duration, measure type (objective or subjective), scoring method, sample size, and follow-up duration.

Most reported outcomes assessed either total physical activity or moderate-to-vigorous physical activity (MVPA). Accordingly, meta-analyses were categorised based on these outcome types. This distinction reflects current physical activity guidelines, which provide recommendations for moderate and vigorous intensity activity rather than light or low-intensity movement [3–5]. As such, it is important to assess intervention effects on MVPA. At the same time, total physical activity offers a broader understanding of participants’ overall activity levels. Outcomes, such as daily step counts or self-reported walking, were not included in the meta-analysis, as too few studies reported these for meaningful synthesis; however, they were included in the narrative summary.

#### Between-group analyses

For studies with a control group that only received usual care, minimal physical activity information, or a non-exercise activity (e.g., stretching), standardised mean differences (SMDs; Hedges’ *g*) were calculated using the metafor package in R. The extracted means, standard deviations, and sample sizes for experimental and control groups at baseline, post-intervention, and long-term follow-up were used to calculate group differences in change scores from baseline to post-intervention and baseline to long-term follow-up. If studies reported multiple follow-up time points, effect sizes were calculated for each.

#### Within-group analyses

Standardised mean change effect sizes (raw score standardisation; SMCR) were calculated using the metafor package in R for studies with no or active control groups, as included studies frequently used these study designs. The extracted means, standard deviations, and sample sizes for each group at baseline, post-intervention, and long-term follow-up were used to calculate change from baseline to post-intervention and baseline to long-term follow-up. If studies reported multiple follow-up time points, effect sizes were calculated for each. The correlations between baseline and follow-up measurements were not reported in any study, so a conservative estimate of *r* = 0.5 was used. Sensitivity analyses were conducted using *r* = 0.3 and *r* = 0.7, with no significant differences in effect sizes. Therefore, *r* = 0.5 was retained for all analyses.

## Results

### Search results

**Fig. 1:**
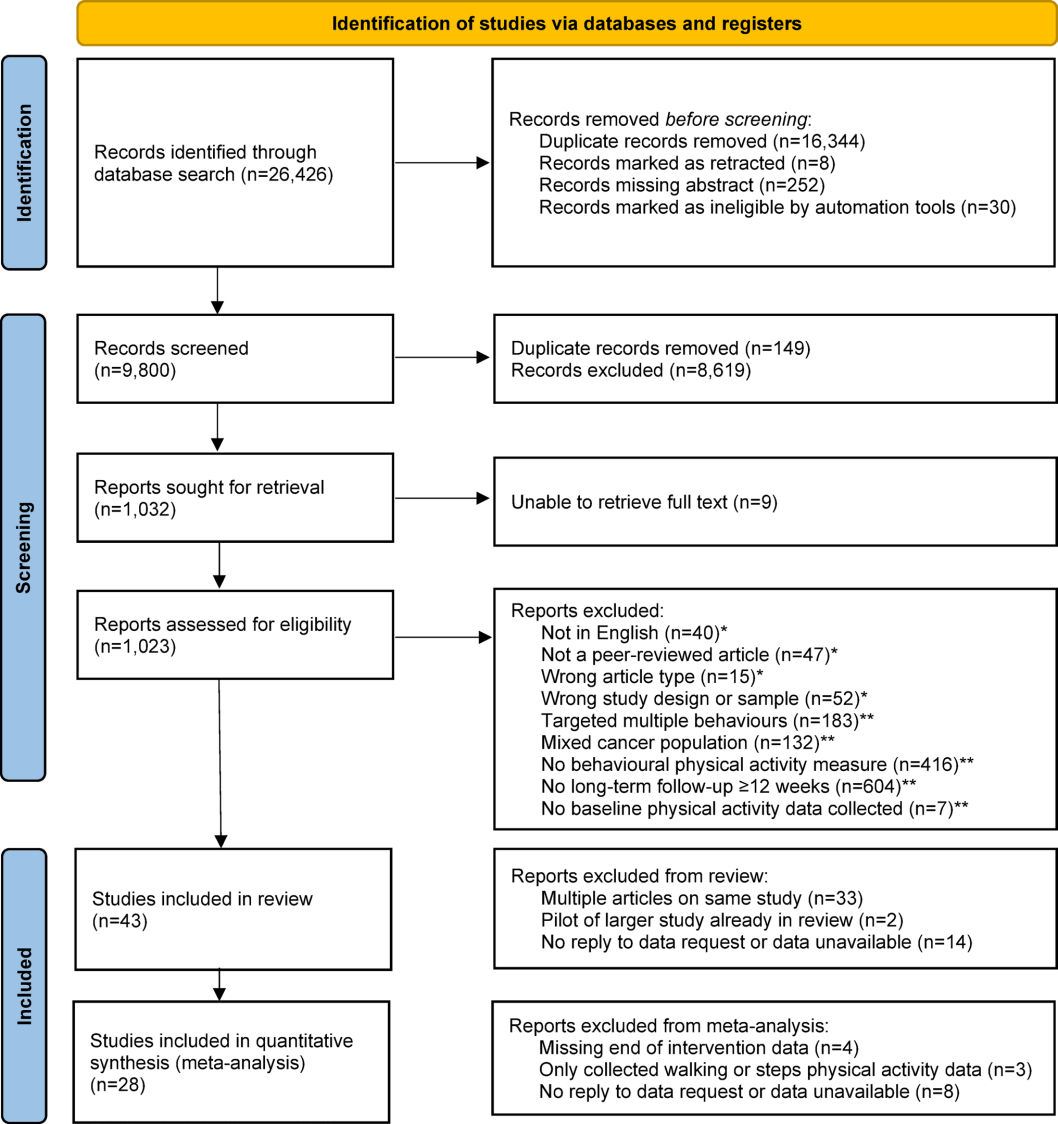
PRISMA flow diagram. Exclusions were applied in two stages. Studies were first screened sequentially for general article criteria (*); if not excluded, they were then screened for breast cancer- and outcome-specific criteria (**)

Database searches identified 26,426 articles. After removing duplicates and ineligible records (e.g., abstracts that were missing, too short, too long, or irrelevant), 9800 abstracts were screened. Approximately 40% (*n* = 4000) of abstracts were manually screened, with *n* = 5795 articles excluded based on Research Screener’s algorithm, and 1032 articles progressing to full-text screening. Corresponding authors (*n* = 10) were contacted via email if articles could not be found; however, only one responded. During the full-text screening, 31 corresponding authors were contacted for additional data, of which ten were provided. From the full-text screening, 43 studies were eligible for inclusion in the review (28 for meta-analysis, 15 for narrative summary only). See Fig. 1 for inclusion of studies at each stage.

### Study characteristics

Included studies are summarised in Table 1. The 43 eligible studies included 5295 participants in total, with a pooled mean age of 54.3 years. Studies were conducted in North America (51.2%, *k* = 22), Europe (27.9%, *k* = 12), Asia (14.0%, *k* = 6), and Australia (7.0%, *k* = 3). Most studies were randomised controlled trials (74.4%, *k* = 32), followed by single-arm designs (18.6%, *k* = 6) and quasi-randomisation (7.0%, *k* = 3). Intervention durations ranged from 3 weeks to 1 year, with 12 weeks being the most common (46.5%, k = 20). Two interventions (4.7%) had flexible durations to align with participants’ chemotherapy cycles but reported mean and median durations of ~ 17 weeks. Long-term follow-up durations ranged from 3 months to 5 years, with 3 months being the most common (48.8%, *k* = 21). Studies tended to use subjective measures (self-report questionnaires) of physical activity levels (60.5%, *k* = 26), followed by objective measures, such as pedometers or accelerometers (23.3%, *k* = 10), and those using both (16.3%, *k* = 7).

Most interventions were with women who had completed adjuvant chemotherapy or radiotherapy prior to participating in the experimental intervention (46.5%, *k* = 20). However, a substantial proportion were with women undergoing adjuvant treatment (27.9%, *k* = 12) or did not exclude by treatment phase (16.3%, *k* = 7). The remaining studies involved women undergoing surgery (4.7%, *k* = 2) or endocrine therapy (4.7%, *k* = 2). Most studies were with women diagnosed with non-metastatic cancer (74.4%, *k* = 32), seven studies did not exclude by cancer stage (16.3%), and 1 was exclusively with women with metastatic cancer (2.3%). The remaining three studies neither mentioned cancer stage in their eligibility criteria nor reported stage data (7.0%).

**Table 1:** Study characteristics

Study ID	*n*	Mean age	Intervention timing	Cancer stage	RCT	Intervention components	Exercise setting	Control group	Duration (wk)	Follow-up (mos.)	Measures	In meta-analysis
Anandavadivelan 2024 (Sweden) [26]	206	53.3 ± 10.3	Chemotherapy	Stage I-IIIa eligible; stage data not reported	Yes	Exercise program	Supervised, face-to-face	Usual care	16	12, 24, 60	ActiGraph GT3X+ accelerometer	No
Ballinger 2021 (USA) [27]	57	59.2 ± 9.4	Completed adjuvant treatments ≥ 4 weeks	Stage 0: *n* = 2 (3.5%) Stage I: *n* = 30 (52.6%) Stage II: *n* = 18 (31.6%) Stage III: *n* = 7 (12.3%)	No	Exercise program Self-monitoring	Unsupervised, home-based	None	12	3	Garmin Vivoactive HR accelerometer	No
Baumann 2017 (Germany) [28]	194	55.7 ± 9.2	Any treatment phase; <5 years post-diagnosis	Stage IV excluded (non-metastatic disease only); stage data not reported	No	Exercise program	Supervised, face-to-face (one-on-one) & unsupervised, home-based	Usual care	32	4, 10, 16	Freiburg Questionnaire on Physical Activity	Yes
Changizi 2022 (Iran) [29]	123	45.7 ± 8.0	Post-surgery	Eligibility criteria does not mention cancer stage; stage data not reported	Yes	Exercise program Relaxation training Education Peer social support	Unsupervised, home-based	Usual care	12	3	Persian IPAQ-Long Form	Yes
Guinan 2013 (Ireland) [30]	26	48.1 ± 8.8	< 6 months post-chemotherapy	Stage I: *n* = 7 (26.9%) Stage II: *n* = 13 (50.0%) Stage III: *n* = 6 (23.1%)	Yes	Exercise program	Supervised, face-to-face (group setting) & unsupervised, home-based	Usual care	8	3	RT3 Activity monitor & GLTEQ	Yes
Han 2023 (South Korea) [31]	46	48.9 ± 7.1	Completed chemotherapy; <5 years post-diagnosis	Stage I: *n* = 12 (26.1%) Stage II: *n* = 26 (56.5%) Stage III: *n* = 8 (17.4%)	Yes	Exercise program Education Peer social support Self-monitoring	Supervised, face-to-face (group-based) & unsupervised, home-based	Usual care	12	6	Korean GPAQ	Yes
Hartman 2022 (USA) [32]	75	57.2 ± 10.4	Completed chemo- or radiotherapy; <5 years post-diagnosis	Stage I: *n* = 44 (58.7%) Stage II: *n* = 24 (32.0%) Stage III: *n* = 7 (9.3%)	Yes	Goal setting session Self-monitoring Coaching support	Unsupervised, home-based	Waitlist control	12	24	ActiGraph GT3X+ accelerometer (pre-intervention) & Fitbit One (intervention & follow-up)	No
Husebø 2014 (Norway) [33]	67	52.2 ± 9.3	Chemotherapy	Stage I: *n* = 19 (28.4%) Stage II: *n* = 34 (56.7%) Stage III: *n* = 7 (11.6%)	Yes	Exercise program Coaching support	Unsupervised, home-based	Usual care	18–24 (*M* = 17.2)	6	IPAQ-Short Form	Yes
Iwamoto 2024 (Japan) [34]	342	55.0 ± 10.9	Completed chemotherapy < 1 year	Stage 0: *n* = 36 (10.5%) Stage I: *n* = 158 (46.2%) Stage II: *n* = 111 (32.5%) Stage III: *n* = 36 (10.5%) Missing: *n* = 1(0.3%)	Yes	Exercise program	Supervised, face-to-face (group setting)	Usual care	16	8	Work and leisure-time PA questionnaire	Yes
Ki-Yong 2020 (Canada) [35]	301	50.0 ± 8.9	Chemotherapy	Stage I: *n* = 101 (33.6%) Stage II: *n* = 169 (56.1%) Stage III: *n* = 31 (10.3%)	Yes	Exercise program	Supervised, face-to-face	All arms received an intervention	12–18 (median: 17)	6, 12, 24	GLTEQ	No
Kong 2021 (South Korea) [36]	152	47.1 ± 8.1	Radiotherapy	Stage I: *n* = 28 (18.4%) Stage II: *n* = 62 (40.8%) Stage III: *n* = 62 (40.8%)	Yes	Self-monitoring Education Coaching support	Unsupervised, home-based	All arms received an intervention	5	3, 6	GPAQ	Yes
Leach 2019 (USA) [37]	26	51.9 ± 8.8	Radiotherapy	Stage I: *n* = 10 (38.5%) Stage II: *n* = 15 (57.7%) Uncertain: *n* = 1 (3.8%)	Yes	Exercise program Education	Supervised, face-to-face (group setting OR one-on-one)	All arms received an intervention	8	3	Polar A300 activity tracker & IPAQ - Short Form	Yes
Loo 2019 (USA) [38]	11	Median: 63 ± 10.2	Completed adjuvant treatments ≥ 6 months	Stage IV excluded (non-metastatic disease only); stage data not reported	No	Exercise program	Supervised, face-to-face (group setting)	None	26	6, 18	GLTEQ	No
Lynch 2019 (Australia) [39]	73	62.0 ± 6.5	Completed adjuvant treatments	Stage I: *n* = 20 (27.4%) Stage II: *n* = 37 (50.7%) Stage III: *n* = 16 (21.9%)	Yes	Goal setting session Self-monitoring Coaching support	Unsupervised, home-based	Waitlist control	12	3	ActiGraph GT3X+ accelerometer	Yes
Mavropalias 2023 (Australia) [40]	89	52.0 ± 10.0	Radiotherapy	Stage IV excluded (non-metastatic disease only); stage data not reported	Yes	Exercise program Coaching support	Unsupervised, home-based	Usual care	12	4, 10	GLTEQ	No
McNeil 2019 (Canada) [41]	45	58.7 ± 9.4	Completed adjuvant treatments	Stage I: *n* = 17 (37.8%) Stage II: *n* = 20 (44.4%) Stage III: *n* = 8 (17.8%)	Yes	Exercise program Self-monitoring Education	Unsupervised, home-based	Usual care	12	3	ActiGraph GT3X+ accelerometer	Yes
Min 2024 (South Korea) [42]	56	50.3 ± 6.6	Post-surgery	Stage 0: *n* = 8 (14.3%) Stage I: *n* = 31 (55.4%) Stage II: *n* = 16 (28.6%) Stage III: *n* = 1 (1.8%)	Yes	Exercise program Self-monitoring	Supervised, face-to-face (one-on-one) & unsupervised, home-based	Usual care	4	5	Korean GPAQ	Yes
Møller 2020 (Denmark) [43]	153	51.7 ± 9.4	Chemotherapy	Stage I: *n* = 56 (36.6%) Stage II: *n* = 81 (52.9%) Stage III: *n* = 16 (10.5%)	Yes	Exercise program Self-monitoring	Supervised, face-to-face (group setting) or unsupervised, home-based	All arms received an intervention	12	6	Self-developed questionnaire	No
Mur-Gimeno 2024 (Spain) [44]	24	55.0 ± 10.1	Completed adjuvant treatments ≥ 3 months	Stage I-II: *n* = 16 (66.7%) Stage III-IV: *n* = 8 (33.3%)	Yes	Exercise program	Supervised, face-to-face (group setting)	All arms received an intervention	12	3	ActiGraph GT3X+ accelerometer	Yes
Mutrie 2012 (Scotland) [45]	201	51.6 ± 9.5	Chemo- or radiotherapy	Stage 0-III eligible; stage data not reported	Yes	Exercise program Education	Supervised, face-to-face (group setting) & unsupervised, home-based	Usual care	12	6, 18, 60	Scottish Physical Activity Questionnaire	Yes
Nyrop 2017 (USA) [46]	62	63.8 ± 8.3	Endocrine therapy (aromatase inhibitor)	Stage I: *n* = 25 (40.3%) Stage II: *n* = 20 (32.3%) Stage III: *n* = 7 (11.3%) Stage IV: *n* = 10 (16.1%)	Yes	Exercise program Self-monitoring Education	Unsupervised, home-based	Waitlist control	6	6	Self-reported walking time	No
Ormel 2021 (Netherlands) [47]	141	61 (range 34–74)	Endocrine therapy	Stage I: *n* = 65 (46.1%) Stage II: *n* = 58 (41.1%) Stage III: *n* = 18 (12.8%)	No	Exercise program	Supervised, face-to-face	None	12	3	GT3XBT accelerometer & PASE questionnaire	Yes
Penttinen 2019 (Finland) [48]	446	53 ± 8	Completed adjuvant treatments ≥ 4 months	Stage IV excluded (non-metastatic disease only); stage data not reported	Yes	Exercise program	Supervised, face-to-face (group setting) & unsupervised, home-based	Usual care	52	60	Unnamed leisure-time physical activity measure (pre-intervention) & 2-week diary (intervention & follow-ups)	No
Phillips 2022 (USA) [49]	269	52.5 ± 9.9	Completed adjuvant treatments ≥ 3 months	Stage I: *n* = 105 (39.0%) Stage II: *n* = 113 (42.0%) Stage III: *n* = 43 (16.0%) Not reported: *n* = 8 (3.0%)	Yes	Exercise program Self-monitoring Education Coaching/peer support	Unsupervised, home-based	All arms received an intervention	12	3	ActiGraph GT3X+ accelerometer	Yes
Pinto 2008 (USA) [50]	86	53.1 ± 9.8	Completed adjuvant treatments; <5 years post-diagnosis	Stage 0: *n* = 14 (16.3%) Stage I: *n* = 32 (37.2%) Stage II: *n* = 40 (46.5%)	Yes	Self-monitoring Education Coaching support	Unsupervised, home-based	Contact control	24	3	7-Day Physical Activity Recall	No
Pinto 2013 (USA) [51]	192	60.0 ± 9.9	Completed adjuvant treatments; <5 years post-diagnosis	Stage 0: *n* = 24 (12.5%) Stage I: *n* = 74 (38.5%) Stage II: *n* = 78 (40.6%) Stage III-IV: *n* = 16 (8.3%)	Yes	Self-monitoring Education Coaching support	Unsupervised, home-based	Contact control	24	6	7-Day Physical Activity Recall	No
Pinto 2015 (USA) [52]	76	55.6 ± 9.5	Completed surgery	Stage 0: *n* = 5 (6.58%) Stage I: *n* = 29 (38.16%) Stage II: *n* = 34 (44.74%) Stage III: *n* = 8 (10.53%)	Yes	Self-monitoring Coaching/peer support	Unsupervised, home-based	Contact control	12	3	ActiGraph GT3X accelerometer & 7-Day Physical Activity Recall	Yes
Pinto 2022 (USA) [53]	161	57.3 ± 10.8	Any treatment phase; <5 years post-diagnosis (non-metastatic)	Stage 0: *n* = 26 (16.1%) Stage I: *n* = 75 (46.6%) Stage II: *n* = 44 (27.3%) Stage III: *n* = 16 (9.9%)	Yes	Self-monitoring Education Coaching support	Unsupervised, home-based	All arms received an intervention	36	3	ActiGraph GT3X accelerometer & 7-Day Physical Activity Recall	Yes
Rabin 2009 (USA) [54]	23	52.5 ± 8.4	Completed adjuvant treatments; <5 years post-diagnosis	Stage 0: *n* = 2 (8.7%) Stage I: *n* = 10 (43.5%) Stage II: *n* = 11 (47.8%)	No	Relaxation training Self-monitoring Coaching support	Unsupervised, home-based	None	12	3	Biotrainer accelerometer	Yes
Rogers 2023 (USA) [55]	222	54.4 ± 8.5	Completed adjuvant treatments	Stage 0: *n* = 25 (11.3%) Stage I: *n* = 93 (41.9%) Stage II: *n* = 78 (35.1%) Stage III: *n* = 26 (11.7%)	Yes	Exercise program Education Coaching support	Supervised, face-to-face	Usual care	12	3, 9	MTI/ActiGraph accelerometer & GLTEQ	Yes
Schmidt 2017 (Germany) [56]	227	54.6 ± 9.4	Chemo- or radiotherapy	Stage 0: *n* = 12 (5.3%) Stage I: *n* = 115 (50.7%) Stage II: *n* = 74 (32.6%) Stage III+: *n* = 26 (11.5%)	Yes	Exercise program	Supervised, face-to-face (group setting)	Jacobson-method relaxation	12	3, 12	Short Questionnaire to Assess Health-enhancing PA [Modified]	Yes
Schulz 2022 (Germany) [57]	19	58.7 ± 7.6	Any treatment phase; <2 years post-diagnosis	Stage IV excluded (non-metastatic disease only); stage data not reported	No	Exercise program	Supervised, face-to-face	Usual care	6	12, 24	Human Activity Profile	Yes
Shachar 2023 (USA) [58]	52	55.0 ± 11.1	Any treatment phase; metastatic only	Stage IV: *n* = 52 (100.0%)	No	Self-monitoring Education Coaching support	Unsupervised, home-based	None	12	3	Self-reported walking time	No
Smith-Turchyn 2020 (Canada) [59]	26	49.0 ± 12.3	Chemotherapy	Stage I: *n* = 2 (7.7%) Stage II: *n* = 12 (46.1%) Stage III: *n* = 12 (46.1%)	Yes	Exercise program Education	Supervised, face-to-face (one-on-one)	Usual care	16	4	GLTEQ	Yes
Soleimani 2016 (Iran) [60]	70	44.5 ± 6.5	Any treatment phase	Eligibility criteria does not mention cancer stage; stage data not reported	No	Education	Unsupervised, home-based	Usual care	4	3	Persian IPAQ - Long Form	No
Soltero 2023 (USA) [61]	104	59.9 ± 8.6	Completed adjuvant treatments ≥ 6 months	Stage 0: *n* = 8 (7.7%) Stage I: *n* = 40 (38.5%) Stage II: *n* = 38 (36.5%) Stage III: *n* = 18 (17.3%)	Yes	Exercise program Education	Supervised, face-to-face (group setting) & unsupervised, home-based	All arms received an intervention	8	7	Women’s Health Initiative Brief Physical Activity Questionnaire	No
Spence 2022 (Australia) [62]	60	50.1 ± 9.0	Any treatment phase; <5 years post-diagnosis	Stage II: *n* = 28 (46.7%) Stage III: *n* = 20 (30.3%) Stage IV: *n* = 7 (11.7%) Unsure: *n* = 5 (8.3%)	Yes	Exercise program Education	Supervised, face-to-face & unsupervised, home-based	All arms received an intervention	12	3	Active Australia Survey	Yes
Vallance 2008 (Canada) [63]	377	58 (range: 30–90)	Completed adjuvant treatments	Stage I: *n* = 194 (51.5%) Stage IIa: *n* = 111 (29.4%) Stage IIb: *n* = 50 (13.3%) Stage III: *n* = 22 (5.8%)	Yes	Self-monitoring Education	Unsupervised, home-based	Usual care	12	6	GLTEQ	Yes
Vani 2024 (Canada) [64]	41	50.7 ± 11.2	Any treatment phase	Stage 0-IV eligible; stage data not reported	No	Exercise program Coaching/peer support	Supervised (remote), home-based	None	8	3	GLTEQ	Yes
Weiner 2023 (USA) [65]	34	43.1 ± 5.5	Completed adjuvant treatments ≥ 6 months	Stage I: *n* = 8 (23.5%) Stage II: *n* = 15 (44.1%) Stage III: *n* = 10 (29.4%) Stage IV: *n* = 1 (2.9%)	No	Exercise program Coaching/peer support	Unsupervised, home-based	None	12	3	ActiGraph GT3X+ accelerometer	Yes
Wilson 2005 (USA) [66]	22	55 (range: 47–66)	Completed adjuvant treatments ≥ 3 months	Eligibility criteria does not mention cancer stage; stage data not reported	No	Self-monitoring Education Peer support	Unsupervised, home-based	None	8	3	Steps-only pedometer	No
Winters-Stone 2022 (USA) [67]	114	70.9 ± 5.1	Completed adjuvant treatments ≥ 2 years	Stage 0: *n* = 14 (12.3%) Stage I: *n* = 54 (47.4%) Stage II: *n* = 34 (29.8%) Stage III: *n* = 9 (7.9%) Missing: *n* = 3 (2.6%)	Yes	Exercise program	Supervised, face-to-face (group setting)	Supervised stretching & relaxation program	52	6	CHAMPS physical activity questionnaire	Yes
Witlox 2018 (Netherlands) [68]	204	49.6 ± 8.1	Chemotherapy	Stage IV excluded (non-metastatic disease only); stage data not reported	Yes	Exercise program	Supervised, face-to-face (group setting)	Usual care	18	4, 44	Short Questionnaire to Assess Health-enhancing PA	Yes

*CHAMPS* Community Health Activity Model Program for Seniors, *GPAQ* Global Physical Activity Questionnaire, *IPAQ* International Physical Activity Questionnaire, *PASE* Physical Activity Scale for the Elderly, *QLTEQ* Godin Leisure Time Exercise Questionnaire

### Risk of bias

The risk of bias assessment highlighted inherent biases of behavioural interventions that can be difficult to control (see Supplemental Material 3 for completed checklists). In all randomised controlled trials, participants and intervention facilitators were not blinded to group allocation. Eleven studies had outcome assessors blinded to participants’ group assignments, 11 reported assessors were not blinded, and 10 did not specify. Groups appeared to be similar at baseline in 7 studies, 6 reported baseline imbalances, and 19 reported no imbalances, but groups had large numerical differences in demographic variables. Most studies (*k* = 23) showed good similarity with the treatments received by participants outside of the experimental interventions; however, two studies showed differences in the breast cancer treatments received by participants: one only included participants undergoing chemotherapy but reported substantial variation in chemotherapy regimens across groups, and the other found the control group was more likely to be receiving hormone therapy and less likely to be partnered compared to the experimental group. Seven studies did not report sufficient information to make a judgment. Outcome measures were validated; however, the delivery method was rarely reported (e.g., via online questionnaire or interviewer-led), making it difficult to ascertain reliability of outcome measurement.

Of the nine quasi-experimental studies, only three included a control group. Of these, two showed differences in the breast cancer treatments participants were receiving outside of the experimental intervention, with the experimental groups more likely to be undergoing chemotherapy compared to the control group. Detailed participant retention information was available for six studies; three studies reported full details for some timepoints or gave an overall account of retention, the remaining two did not report these details (*k* = 1), or the data could not be accessed (*k* = 1). As with the randomised controlled trials, few studies reported how outcome measures were administered.

### Meta-analyses

#### Between-group analyses

**Fig. 2:**
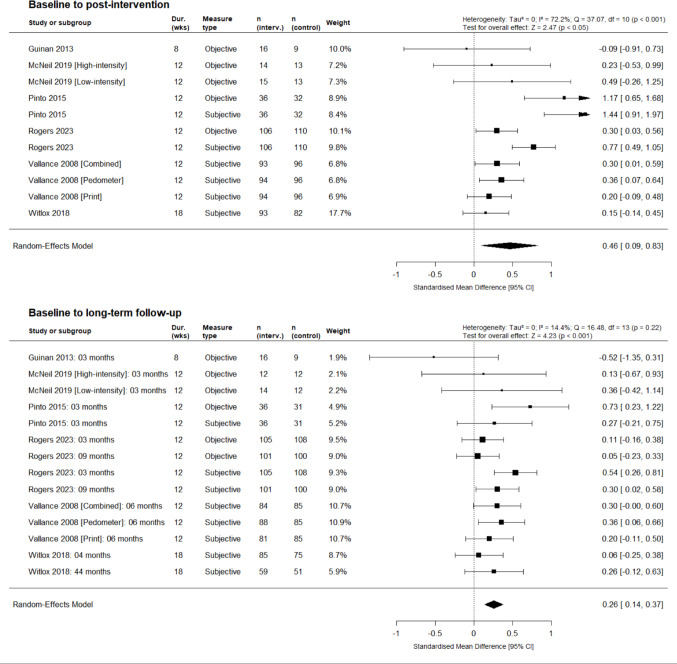
Between-group differences in change in moderate-to-vigorous physical activity

Six studies included MVPA outcomes, contributing 11 effect sizes from baseline to post-intervention, and 14 from baseline to long-term follow-up (Fig. 2). From baseline to post-intervention, experimental groups showed greater increases in MVPA compared to control groups with a moderate effect size (SMD = 0.46, 95% CI [0.09,0.83]); with moderate to high heterogeneity: *Q*(10) = 37.07, *p*<0.001, *I*^2^_total_=72.2. Experimental groups maintained greater changes from baseline at long-term follow-up compared to control groups with a small effect size (SMD = 0.26, [0.14, 0.37]). Heterogeneity was low and non-significant: *Q*(13) = 16.48, *p* = 0.224). Egger’s test of publication bias was non-significant for both analyses (*p* = 0.989 and 0.671, respectively).

**Fig. 3:**
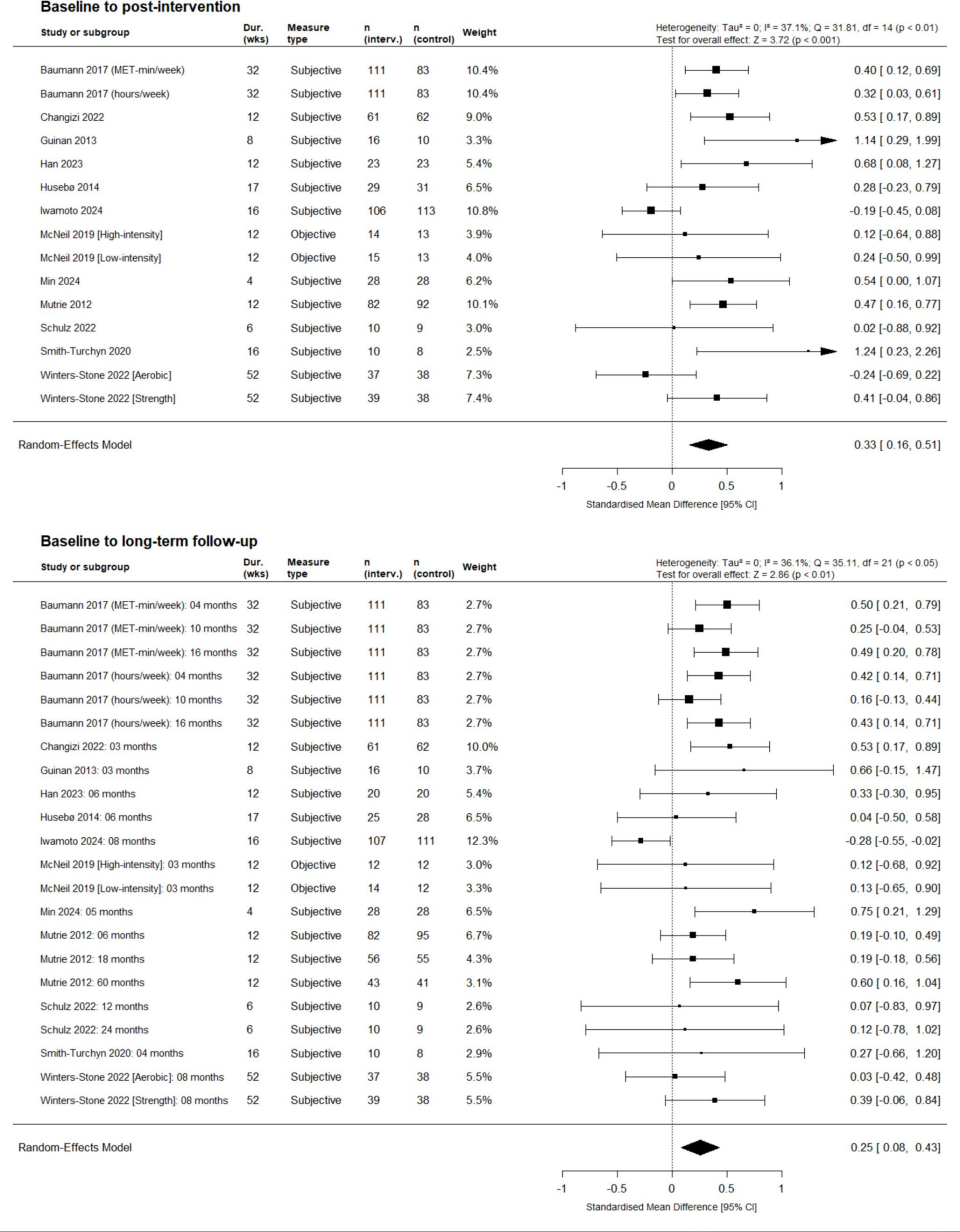
Between-group differences in change in total physical activity

Twelve studies included total physical activity outcomes, contributing 17 effect sizes from baseline to post-intervention, and 22 from baseline to long-term follow-up (Fig. 3). From baseline to post-intervention, experimental groups showed greater increases in total physical activity compared to control groups with a small-to-moderate effect size (SMD = 0.33, [0.16,0.51]); with moderate heterogeneity: *Q*(14) = 31.81, *p* = 0.004, *I*^2^_total_=37.1. Experimental groups maintained greater changes from baseline at long-term follow-up compared to control groups with a small effect size (SMD = 0.25, [0.08, 0.43]); with moderate heterogeneity: *Q*(21) = 35.11, *p*=0.027, *I*^2^_total_=36.1. Egger’s tests of publication bias were non-significant (*p* = 0.140 and 0.475, respectively).

Exploratory moderator analyses were only significant for MVPA from baseline to post-intervention, with measure type being a significant moderator (*p* = 0.021). Effect sizes were larger in studies using subjective measures compared to objective ones (β = 0.38, [0.06, 0.71]); however, heterogeneity was higher than in the base model: *Q*(9) = 37.07, *p* = 0.002, *I*^2^_total_=76.8.

#### Within-group analyses

**Fig. 4:**
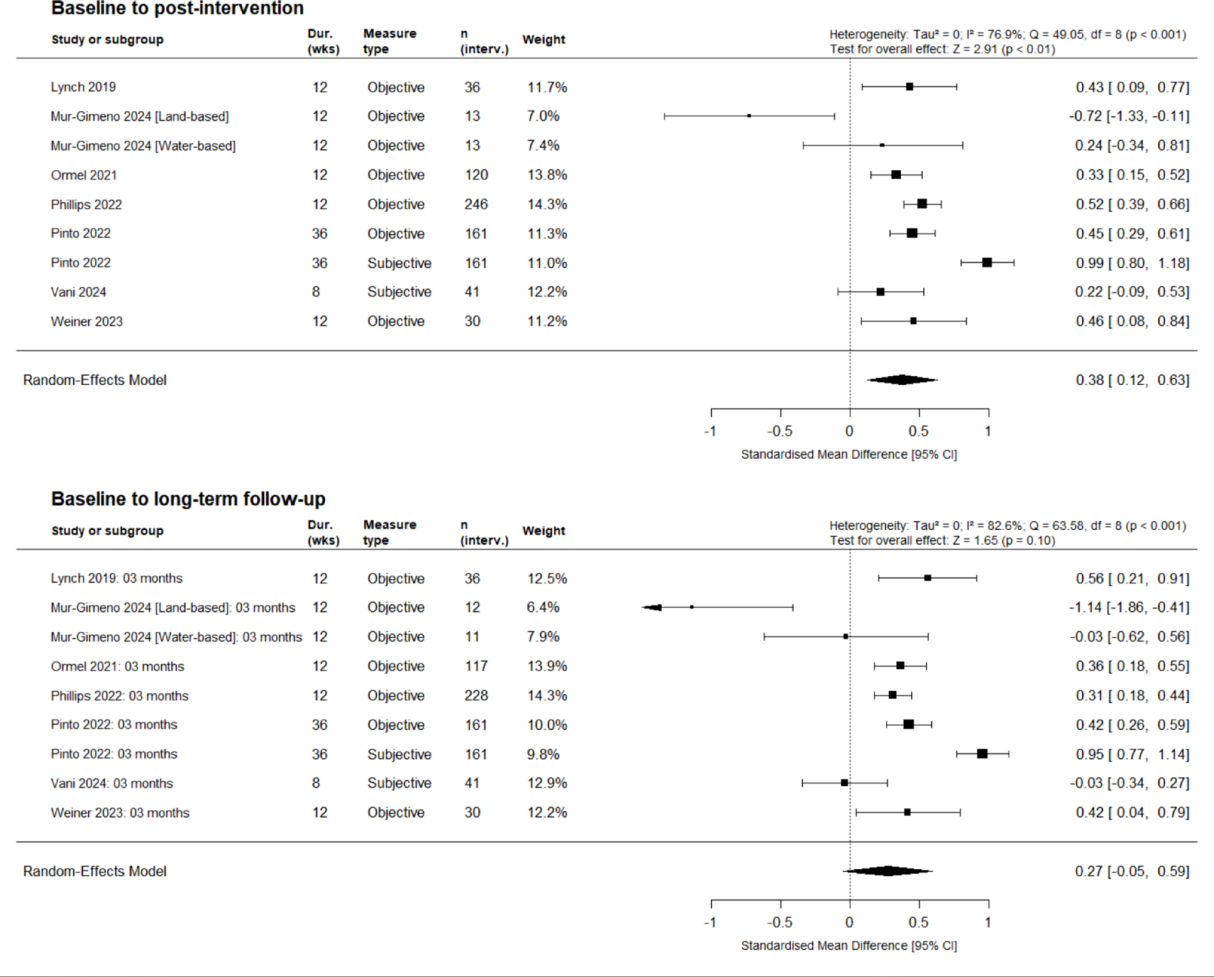
Within-group changes in moderate-to-vigorous physical activity

Seven studies included MVPA outcomes, contributing 9 effect sizes from baseline to post-intervention, and from baseline to long-term follow-up (Fig. 4). From baseline to post-intervention, participants increased their MVPA with a small-to-moderate effect size (SMD = 0.38, [0.12,0.63]); with high heterogeneity: *Q*(8) = 49.05, *p* < 0.001, *I*^2^_total_=76.9. MVPA remained higher than baseline at long-term follow-up; however, changes were not statistically significant (SMD = 0.27, [− 0.05, 0.59]); with high heterogeneity: *Q*(8) = 63.58, *p*< 0.001, *I*^2^_total_=82.6. Egger’s test of publication bias was significant in both analyses (*p* = 0.014 and 0.006, respectively), suggesting that smaller studies trended towards larger effect sizes.

**Fig. 5:**
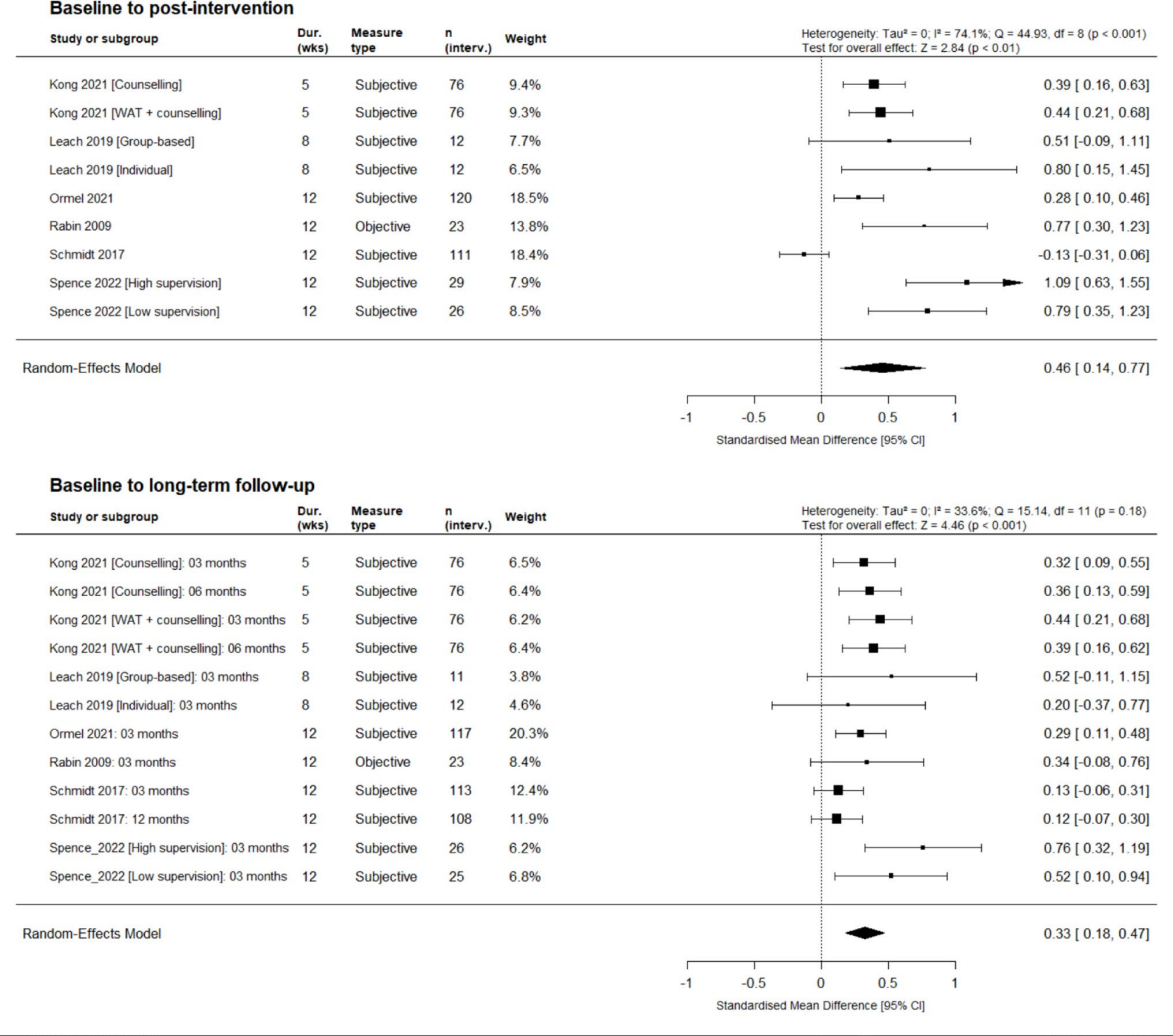
Within-group changes in total physical activity

Six studies included total physical activity outcomes, contributing 9 effect sizes from baseline to post-intervention, and 12 from baseline to long-term follow-up (Fig. 5). From baseline to post-intervention, participants increased their total physical activity with a moderate effect size (SMD = 0.46, [0.14, 0.77]); with high heterogeneity: *Q*(8) = 44.93, *p*<0.001, *I*^2^_total_=74.1. Total physical activity remained higher than baseline at long-term follow-up (SMD = 0.33, [0.18, 0.47]); with moderate heterogeneity: *Q*(11) = 15.14, *p*<0.001, *I*^2^_total_=33.6. Egger’s test of publication bias was significant in the baseline to post-intervention analysis only (*p* = 0.014), suggesting that smaller studies trended towards larger effect sizes for that timepoint.

Exploratory moderator analyses were significant for total physical activity from baseline to post-intervention, with sample size being a significant moderator. Studies with larger sample sizes showed smaller effect sizes (β=− 0.007, [− 0.01, − 0.00]), with a moderate reduction in heterogeneity compared to the base model: Q(7) = 16.45, *p*=0.021, *I*^2^_total_=44.7.

### Narrative summary of articles not included in meta-analyses

Studies were excluded from meta-analyses due to missing means and standard deviations (*k* = 8), not collecting post-intervention outcome data (*k* = 4), or only collecting steps or self-reported walking data (*k* = 3). Characteristics of these studies are summarised in Table 1.

Three single-arm studies only measured walking. Two showed increases and maintenance of steps and self-reported walking time from baseline to three months post-intervention [58, 66], while the third found that only about a third of participants maintained daily step counts at follow-up [27]. A fourth wait-list controlled study assessed walking, and found significant post-intervention gains for the experimental group, but these declined by six-month follow-up, when both groups had received the intervention [46]. A cultural dance intervention also found significant increases in moderate-intensity physical activity that were maintained six- and twelve-months post-intervention [38]. However, no significant differences in vigorous physical activity were found.

For the controlled studies, immediate post-intervention effects were mixed, with greater improvements reported in the experimental arms for two studies [32, 51], while another found no significant differences between experimental and control arms [48]. One study reported a temporary decline in activity at six months, while participants were still receiving monthly calls [50]. Another found that, although the experimental group maintained significant increases in MVPA up to twelve months post-intervention, their activity levels did not differ significantly from the control group [40]. In a comparison of two active experimental arms (supervised exercise and unsupervised home-based walking), both groups significantly increased MVPA; however, the supervised group demonstrated significantly greater gains in higher-intensity physical activity [43]. In contrast, a study comparing supervised exercise with an education-only arm found no significant between-group differences in objectively or subjectively measured physical activity at any timepoint [61]. Of the studies that either demonstrated short-term improvements or did not collect immediate post-intervention measures, three studies reported that physical activity levels returned to baseline [26, 32, 51], and three showed sustained or increased activity [35, 50, 60]. Follow-up duration varied from three months to five years post-intervention, but there was no clear indication of longer durations being associated with larger decreases in physical activity.

## Discussion

This review evaluated the short- and long-term effectiveness of physical activity interventions for women living with and beyond breast cancer. Findings suggest that interventions effectively increase total and moderate-to-vigorous physical activity when participants are actively supported, with effect sizes ranging from small to moderate across analyses. However, the long-term effects are unclear. In the between-group analyses, studies measuring MVPA had larger between-group difference effect sizes post-intervention, compared to those measuring total physical activity. However, MVPA effects diminished at long-term follow-up, whereas the total physical activity studies remained more consistent. Similarly, previous reviews report greater decreases in the difference between experimental groups and controls for MVPA over time compared to total physical activity [9, 13]. Such results might suggest that participants increase the intensity of their physical activity during more supportive and educational interventions, for example, through tailored exercise education and supports. As active intervention support ceases, higher-intensity physical activity reduces, yet overall physical activity levels are maintained. Alternatively, higher-intensity physical activity may be more readily abandoned post-intervention as a newer and more complex behaviour that is not yet habitual, compared to total physical activity, which may reflect a longer-standing behaviour more resilient to change [69, 70]. These patterns align with within-group findings, with studies measuring total physical activity showing slightly larger mean change effect sizes post-intervention compared to those measuring MVPA. Both groups of studies showed similar decreases in effect sizes at long-term follow-up; however, long-term mean MVPA change from baseline was not statistically significant.

Publication bias may be a concern for the within-group analyses, with Egger’s tests being significant in three of four analyses, suggesting smaller studies are contributing larger effect sizes. This could be due to some single-arm pilot or feasibility studies, which may be more likely to be published when results are promising, potentially overrepresenting positive findings [71]. Significant heterogeneity was also observed across most models, which limits confidence in the pooled estimates. However, this level of heterogeneity is consistent with systematic reviews of physical activity interventions [9, 11, 14] and likely reflects true variation in intervention characteristics, such as duration, delivery modality, participant populations, breast cancer stage and treatment status, and outcome measurement. These findings underscore the complexity of designing and evaluating physical activity interventions for women living with and beyond breast cancer. While several models showed statistically significant changes, the lower bounds of the confidence intervals were often small, suggesting that true effects may be minimal in some cases. This may reflect the wide variability in physical activity levels between studies, as many studies reported large standard deviations. The substantial within-group variability, seen across both subjective and objective measures, highlights the challenges of measuring and detecting behavioural changes in physical activity.

Exploratory moderation analyses identified two significant moderators: measurement type for MVPA at post-intervention in the between-group analyses, and sample size for total physical activity at post-intervention in the within-group analyses. The between-group MVPA post-intervention results suggest that studies using subjective measures tended to yield larger effects than those using objective measures, consistent with a Cochrane review demonstrating subjective measures were more likely to identify long-term maintenance of physical activity, while objective measures tended to show declines [9]. Self-reported measures may be subject to acquiescence and expectancy biases, particularly in unblinded trials, which could contribute to inflated effect sizes [72]. Similarly, the within-group post-intervention total physical activity findings reflected the publication bias results, with smaller sample sizes associated with larger effects. The lack of other significant moderators may reflect the limited number of available effect sizes, which could have reduced statistical power [73].

By focusing specifically on interventions for women living with and beyond breast cancer, this review provides insight into behaviour change patterns within a population experiencing unique treatment side effects, survivorship experiences, and support needs [12]. Most interventions appear effective at increasing short-term physical activity levels, particularly while participants receive active support. Although declines are common at follow-up, some maintenance persists, suggesting interventions may support ongoing physical activity. These findings suggest a need to better understand the behavioural mechanisms operating during the intervention phase, including the most effective support elements and how women can be more effectively transitioned to maintaining physical activity independently once structured support is withdrawn, particularly during recovery from breast cancer treatments that disrupt routine activity.

Using a multilevel model helped to ameliorate the small number of studies in some analyses [10, 17], allowing for a more nuanced allowing the inclusion of multiple data points from the same study while accounting for the non-independent effects [15]. This approach enabled comparisons between subjective and objective measures in studies that reported both, with comparisons indicating meaningfully different estimates of intervention effects. It also showed that long-term declines in physical activity are not strictly tied to follow-up duration. In several cases, substantial reductions occurred within three to four months post-intervention, while other studies showed increases or fluctuations even after extended follow-up periods. These patterns suggest that behavioural changes may occur soon after support ends, reinforcing the need to examine the transitional period between active intervention and independent maintenance. Qualitative studies of physical activity interventions for individuals living with other types of cancer show that a sudden loss of structure and accountability in behavioural programs may disrupt the transition to independent physical activity [74, 75], highlighting the potential value of incorporating structured physical activity support into routine breast cancer aftercare and survivorship services.

## Limitations

This review has several limitations. The included studies were limited to published articles, which introduces the potential for publication bias, as studies with positive findings are more likely to be published. Smaller studies also tended to report larger effect sizes, which may have inflated the overall estimates. Although studies were weighted using inverse-variance methods in the meta-analysis, this does not fully mitigate small-study effects.

Substantial heterogeneity was observed across studies, reflecting differences in intervention characteristics, participant populations, and outcome measures, which may limit confidence in the pooled estimates. Some of this variation may be due to differences in how physical activity was measured, particularly the frequent use of self-reported measures, which may have introduced bias and contributed to differences in effect sizes. In addition, moderator analyses were limited by the small number of studies and effect sizes, which may reduce confidence in these findings.

Abstract screening was supported by Research Screener, which may have resulted in relevant studies being missed. This risk was mitigated by using initial seed articles, screening until two consecutive blocks contained no eligible studies, and checking the reference lists of included articles and relevant reviews for potential missed articles. Data extraction was also supported by AI-powered tools; however, all extracted data were independently verified by two reviewers, reducing the likelihood of errors.

## Conclusion

Physical activity interventions for women living with and beyond breast cancer are effective in increasing activity levels in the short term. However, longer-term effects are varied, especially for MVPA. These findings highlight the need for sustained support beyond the intervention period. Future research should focus on identifying factors, including psychological determinants, that influence the maintenance of physical activity to support long-term survivorship outcomes, such as promoting psychosocial wellbeing and optimising residual vitality.

## Supplementary Information

Below is the link to the electronic supplementary material.

Supplementary file1

Supplementary file2

Supplementary file3
