# Supplementary material for: Short- and long-term effectiveness of physical activity interventions for women living with and beyond breast cancer: a systematic review and meta-analysis
Source: Breast Cancer. 2026 Apr 27;33(4):801–19. doi: 10.1007/s12282-026-01859-y (PMC13283194; doi:10.1007/s12282-026-01859-y)
Supplement: Supplementary file 1 — Supplementary file1 [file 12282_2026_1859_MOESM1_ESM.docx]

# Supplemental Material 1: Search strategies

## Table S1: Concepts and keywords

|  | ***CONCEPT 1*** | ***CONCEPT 2*** | ***CONCEPT 3*** | | ***CONCEPT 4*** |
| --- | --- | --- | --- | --- | --- |
| **Keywords** | physical activity  exercise  resistance exercise/training  aerobic exercise/training  sports  athletics | breast cancer  breast tumour  malignant  neoplasm  carcinoma | trial  intervention  rehabilitation  program  coaching  promoting  experimental  pilot | follow-up  maintenance  sustaining  long-term  post  continuation  evaluation  outcome  feasibility | |

*Note.* Concept 4 was combined with Concept 3 using ‘or’ functions, while other concepts were combined with ‘and’ functions, i.e., final search strategy was: Concept 1 AND Concept 2 AND (Concept 3 OR Concept 4). Keywords and subject headings within each category were combined with ‘or’ functions.

## Table S2: Medline (Ovid) search strategy

|  | ***CONCEPT 1*** | ***CONCEPT 2*** | ***CONCEPT 3 or CONCEPT 4*** | |
| --- | --- | --- | --- | --- |
| **Search Terms** | ("physical activit*" OR exerci* OR ((resistance OR aerobic OR cardio*) ADJ2 (exerci* OR training)) OR sport* OR athlet*).ti,ab. | (breast* ADJ3 (cancer* OR tumour* OR tumor* OR malignan* OR neoplasm* OR carcinoma*)).ti,ab. | (trial OR interven* OR rehab* OR program* OR coach* OR promot* OR experimen* OR pilot OR "follow-up" OR followup OR "follow up" OR maintenance* OR maintain* OR sustain* OR "long-term" OR "long term" OR longterm OR post* OR continu* OR evaluat* OR outcome* OR feasib* OR adher*).ti,ab. | |
| **Subject Headings (MeSH)** | exp Exercise/ | exp Breast Neoplasms/ |  | Follow-Up Studies/ |

*Note.* Limiter used: ‘Journal articles’

## Table S3: PsycInfo (Ovid) search strategy

|  | ***CONCEPT 1*** | ***CONCEPT 2*** | ***CONCEPT 3 or CONCEPT 4*** | |
| --- | --- | --- | --- | --- |
| **Search Terms** | ("physical activit*" OR exerci* OR ((resistance OR aerobic OR cardio*) ADJ2 (exerci* OR training)) OR sport* OR athlet*).ti,ab. | (breast* ADJ3 (cancer* OR tumour* OR tumor* OR malignan* OR neoplasm* OR carcinoma*)).ti,ab. | (trial OR interven* OR rehab* OR program* OR coach* OR promot* OR experimen* OR pilot OR "follow-up" OR followup OR "follow up" OR maintenance* OR maintain* OR sustain* OR "long-term" OR "long term" OR longterm OR post* OR continu* OR evaluat* OR outcome* OR feasib* OR adher*).ti,ab. | |
| **Subject Headings** | exp Physical Activity/ | exp Breast Neoplasms/ | exp Intervention/ or exp Program Development/ or exp Program Evaluation/ |  |

*Note.* Limiter used: ‘Journal articles’

## Table S4: CINAHL Ultimate (EBSCO) search strategy

|  | ***CONCEPT 1*** | ***CONCEPT 2*** | ***CONCEPT 3 or CONCEPT 4*** | |
| --- | --- | --- | --- | --- |
| **Search Terms** | "physical activit*" OR exerci* OR ((resistance OR aerobic OR cardio*) N2 (exerci* OR training)) OR sport* OR athlet* | breast* N3 (cancer* OR tumour* OR tumor* OR malignan* OR neoplasm* OR carcinoma*) | trial OR interven* OR rehab* OR program* OR coach* OR promot* OR experimen* OR pilot OR "follow-up" OR followup OR "follow up" OR maintenance* OR maintain* OR sustain* OR "long-term" OR "long term" OR longterm OR post* OR continu* OR evaluat* OR outcome* OR feasib* OR adher* | |
| **Subject Headings** | (MH "Physical Therapy+") OR (MH "Exercise+") | (MH "Breast Neoplasms+") | (MH "Intervention Trials") OR (MH "Randomized Controlled Trials+") OR (MH "Community Programs") OR (MH "Community Trials") OR (MH "Nonrandomized Trials") |  |

*Note.* Limiter used: ‘Academic journals’

## Table S5: Scopus (Elsevier) search strategy

|  | ***CONCEPT 1*** | ***CONCEPT 2*** | ***CONCEPT 3 or CONCEPT 4*** |
| --- | --- | --- | --- |
| **Search Terms** | "physical activit*" OR exerci* OR ((resistance OR aerobic OR cardio*) W/2 (exerci* OR training)) OR sport* OR athlet* | breast* W/3 (cancer* OR tumour* OR tumor* OR malignan* OR neoplasm* OR carcinoma*) | trial OR interven* OR rehab* OR program* OR coach* OR promot* OR experimen* OR pilot OR "follow-up" OR followup OR "follow up" OR maintenance* OR maintain* OR sustain* OR "long-term" OR "long term" OR longterm OR post* OR continu* OR evaluat* OR outcome* OR feasib* OR adher* |

*Note.* Limiter used: ‘Article’

## Table S6: Web of Science (Clarivate) search strategy

|  | ***CONCEPT 1*** | ***CONCEPT 2*** | ***CONCEPT 3 or CONCEPT 4*** |
| --- | --- | --- | --- |
| **Search Terms** | (TI=("physical activit*" OR exerci* OR ((resistance OR aerobic OR cardio*) NEAR/2 (exerci* OR training)) OR sport* OR athlet*)) OR AB=("physical activit*" OR exerci* OR ((resistance OR aerobic OR cardio*) NEAR/2 (exerci* OR training)) OR sport* OR athlet*) | (TI=(breast* NEAR/3 (cancer* OR tumour* OR tumor* OR malignan* OR neoplasm* OR carcinoma*))) OR AB=(breast* NEAR/3 (cancer* OR tumour* OR tumor* OR malignan* OR neoplasm* OR carcinoma*)) | (TI=(trial OR interven* OR rehab* OR program* OR coach* OR promot* OR experimen* OR pilot OR "follow-up" OR followup OR "follow up" OR maintenance* OR maintain* OR sustain* OR "long-term" OR "long term" OR longterm OR post* OR continu* OR evaluat* OR outcome* OR feasib* OR adher*)) OR AB=( trial OR interven* OR rehab* OR program* OR coach* OR promot* OR experimen* OR pilot OR "follow-up" OR followup OR "follow up" OR maintenance* OR maintain* OR sustain* OR "long-term" OR "long term" OR longterm OR post* OR continu* OR evaluat* OR outcome* OR feasib* OR adher*) |

*Note.* Limiter used: ‘Article’

## Table S7: ProQuest search strategy

|  | ***CONCEPT 1*** | ***CONCEPT 2*** | ***CONCEPT 3*** | ***CONCEPT 4*** |
| --- | --- | --- | --- | --- |
| **Search Terms** | "physical activit*" OR exerci* OR ((resistance OR aerobic OR cardio*) NEAR/2 (exerci* OR training)) OR sport* OR athlet* | breast* NEAR/3 (cancer* OR tumour* OR tumor* OR malignan* OR neoplasm* OR carcinoma*) | trial OR interven* OR rehab* OR program* OR coach* OR promot* OR experimen* OR pilot OR "follow-up" OR followup OR "follow up" OR maintenance* OR maintain* OR sustain* OR "long-term" OR "long term" OR longterm OR post* OR continu* OR evaluat* OR outcome* OR feasib* OR adher* | |
| **Thesaurus** | MAINSUBJECT.EXACT("Physical activity") OR MAINSUBJECT.EXACT("Exercise") | MAINSUBJECT.EXACT("Breast cancer") |  | MAINSUBJECT.EXACT("Feasibility") OR MAINSUBJECT.EXACT("Feasibility studies") |

*Note.* Limiter used: ‘Peer-reviewed, scholarly journals
