# Supplementary figures and images for: Short- and long-term effectiveness of physical activity interventions for women living with and beyond breast cancer: a systematic review and meta-analysis
Source: Breast Cancer. 2026 Apr 27;33(4):801–19. doi: 10.1007/s12282-026-01859-y (PMC13283194; doi:10.1007/s12282-026-01859-y)

# Title and Abstract Screening

## Inclusion and Exclusion Criteria

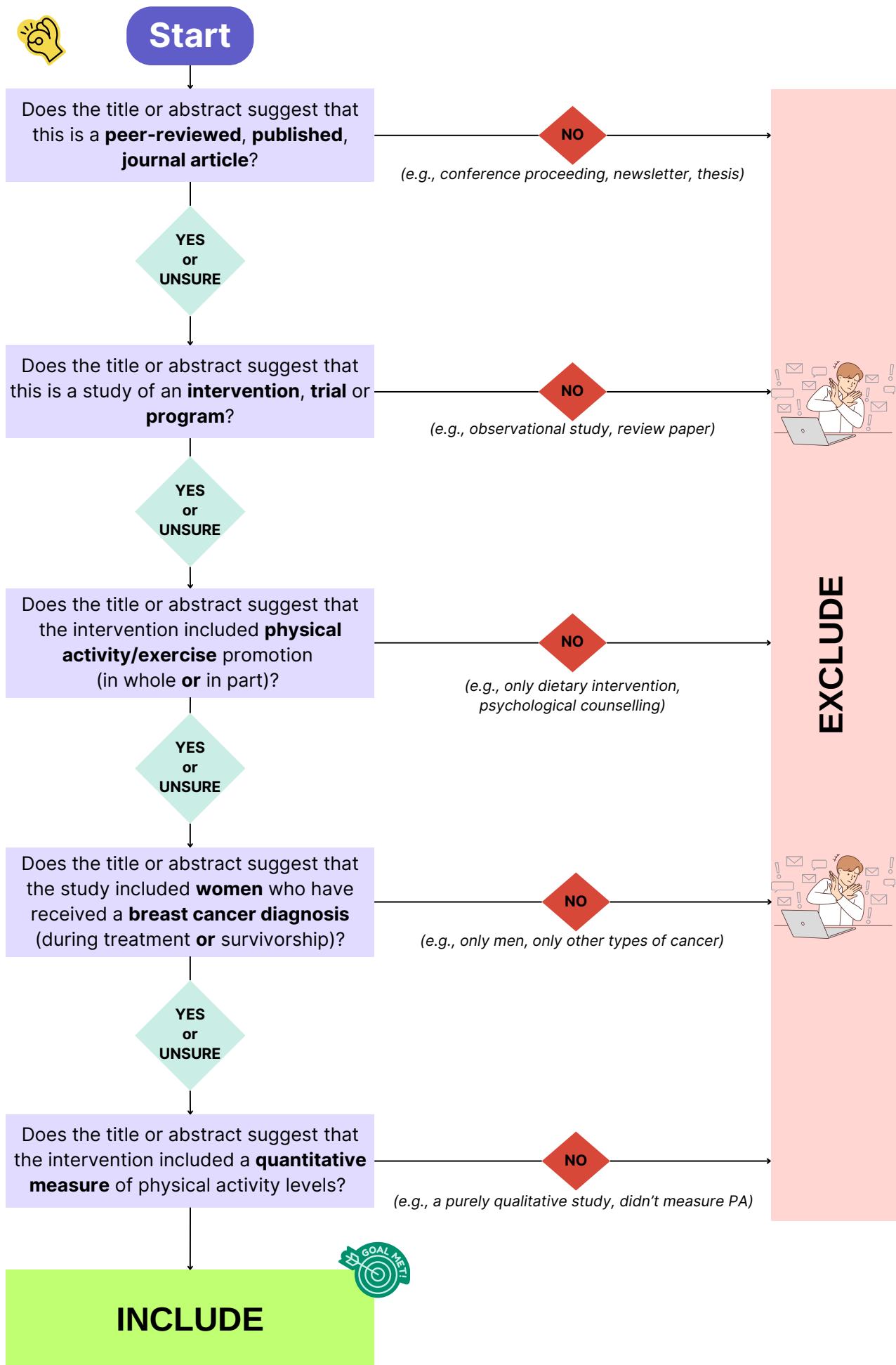

Supplement: Supplementary file 2 — Supplementary file2 [file 12282_2026_1859_MOESM2_ESM.pdf]
