# Supplementary material for: Short- and long-term effectiveness of physical activity interventions for women living with and beyond breast cancer: a systematic review and meta-analysis
Source: Breast Cancer. 2026 Apr 27;33(4):801–19. doi: 10.1007/s12282-026-01859-y (PMC13283194; doi:10.1007/s12282-026-01859-y)
Supplement: Supplementary file 3 — Supplementary file3 [file 12282_2026_1859_MOESM3_ESM.docx]

# Supplemental Material 2: Risk of Bias (JBI) Checklists

| **Table S8.** Risk of bias assessment (randomised controlled trials) | | | | | | | | | | | | | | | |
| --- | --- | --- | --- | --- | --- | --- | --- | --- | --- | --- | --- | --- | --- | --- | --- |
|  |  | **Domain** | **Selection and allocation** | | | **Administration of intervention/exposure** | | | **Assessment, detection, and measurement of outcome** | | | **Participant retention** | **Statistical Conclusion Validity** | | |
| **Study** | **Outcome** | **Result** | **1** | **2** | **3** | **4** | **5** | **6** | **7** | **8** | **9** | **10** | **11** | **12** | **13** |
| Anandavadivelan et al. 2024 | Objective MVPA | 24-months | ✔️ | ✔️ | Unclear | ❌ | ❌ | ✔️ | ❌ | ✔️ | ✔️ | ✔️ | ✔️ | ✔️ | ✔️ |
|  |  | 60-months |  |  |  |  |  |  |  |  |  | ✔️ | ✔️ | ✔️ |  |
| Changizi et al. 2022 | Subjective TPA | Post-intervention | Unclear | ❌ | ❌ | ❌ | ❌ | Unclear | Unclear | ✔️ | Unclear | ❌ | ❌ | Unclear | Unclear |
|  |  | 3-months |  |  |  |  |  |  |  |  |  | ❌ | ❌ | Unclear |  |
| Guinan et al. 2013 | Objective MVPA | Post-intervention | ✔️ | Unclear | Unclear | ❌ | ❌ | ✔️ | ✔️ | ✔️ | Unclear | ✔️ | ✔️ | ✔️ | ✔️ |
|  |  | 3-months |  |  |  |  |  |  |  |  |  | ✔️ | ✔️ | ✔️ |  |
|  | Subjective TPA | Post-intervention |  |  |  |  |  |  | ✔️ | ✔️ | Unclear | ✔️ | ✔️ | ✔️ |  |
|  |  | 3-months |  |  |  |  |  |  |  |  |  | ✔️ | ✔️ | ✔️ |  |
| Han et al. 2023 | Subjective TPA | Post-intervention | ✔️ | ✔️ | Unclear | ❌ | ❌ | ✔️ | ❌ | ✔️ | Unclear | ✔️ | Unclear | ✔️ | ✔️ |
|  |  | 6-months |  |  |  |  |  |  |  |  |  | ✔️ | Unclear | ✔️ |  |
| Hartman et al. 2022 | Objective MVPA | Post-intervention | ✔️ | Unclear | ✔️ | ❌ | ❌ | ✔️ | Unclear | ✔️ | ❌ | ✔️ | ✔️ | ✔️ | ✔️ |
|  |  | 24-months |  |  |  |  |  |  |  |  |  | ✔️ | ✔️ | ❌ |  |
| Husebø et al. 2014 | Subjective TPA | Post-intervention | ✔️ | ✔️ | Unclear | ❌ | ❌ | ✔️ | Unclear | ✔️ | Unclear | ✔️ | Unclear | ✔️ | ✔️ |
|  |  | 6-months |  |  |  |  |  |  |  |  |  | ✔️ | Unclear | ✔️ |  |
| Iwamoto et al. 2024 | Subjective TPA | Post-intervention | Unclear | Unclear | Unclear | ❌ | ❌ | ✔️ | Unclear | ✔️ | Unclear | Unclear | ✔️ | ✔️ | ✔️ |
|  |  | 8-months |  |  |  |  |  |  |  |  |  | Unclear | ✔️ | ✔️ |  |
| Ki-Yong et al. 2020 | Subjective MVPA | 6-months | ✔️ | ✔️ | Unclear | ❌ | ❌ | ❌ | ❌ | ✔️ | ✔️ | ❌ | ✔️ | ✔️ | ✔️ |
|  |  | 12-months |  |  |  |  |  |  |  |  |  | ❌ | ✔️ | ✔️ |  |
|  |  | 24-months |  |  |  |  |  |  |  |  |  | ❌ | ✔️ | ✔️ |  |
| Kong et al. 2021 | Subjective TPA | Post-intervention | ✔️ | ✔️ | Unclear | ❌ | ❌ | Unclear | ✔️ | ✔️ | Unclear | ❌ | ✔️ | ✔️ | ✔️ |
|  |  | 3-months |  |  |  |  |  |  |  |  |  | ❌ | ✔️ | ✔️ |  |
|  |  | 6-months |  |  |  |  |  |  |  |  |  | ❌ | ✔️ | ✔️ |  |
| Leach et al. 2019 | Subjective TPA | Post-intervention | ✔️ | ✔️ | Unclear | ❌ | ❌ | ✔️ | ❌ | ✔️ | Unclear | ✔️ | ❌ | ✔️ | ✔️ |
|  |  | 3-months |  |  |  |  |  |  |  |  |  | Unclear | ❌ | ✔️ |  |
| Lynch et al. 2019 | Objective MVPA | Post-intervention | ✔️ | ✔️ | Unclear | ❌ | ❌ | ✔️ | ❌ | ✔️ | ✔️ | ✔️ | ✔️ | ✔️ | ✔️ |
|  |  | 3-months |  |  |  |  |  |  |  |  |  | ✔️ | ✔️ | ✔️ |  |
| Mavropalias_2023 | Subjective TPA | Post-intervention | Unclear | ✔️ | Unclear | ❌ | ❌ | ✔️ | Unclear | ✔️ | Unclear | ✔️ | ✔️ | ✔️ | ✔️ |
|  |  | 4-months |  |  |  |  |  |  |  |  |  | ✔️ | ✔️ | ✔️ |  |
|  |  | 10-months |  |  |  |  |  |  |  |  |  | ✔️ | ✔️ | ✔️ |  |
| McNeil et al. 2019 | Objective MVPA | Post-intervention | Unclear | Unclear | Unclear | ❌ | ❌ | ✔️ | Unclear | ✔️ | ✔️ | ✔️ | ✔️ | ✔️ | ✔️ |
|  |  | 3-months |  |  |  |  |  |  |  |  |  | ✔️ | ✔️ | ✔️ |  |
|  | Objective TPA | Post-intervention |  |  |  |  |  |  | Unclear | ✔️ | ✔️ | ✔️ | ✔️ | ✔️ |  |
|  |  | 3-months |  |  |  |  |  |  |  |  |  | ✔️ | ✔️ | ✔️ |  |
| Min et al. 2024 | Subjective TPA | Post-intervention | ✔️ | ✔️ | ✔️ | ❌ | ❌ | ✔️ | ❌ | ✔️ | Unclear | ✔️ | ✔️ | ✔️ | ✔️ |
|  |  | 5-months |  |  |  |  |  |  |  |  |  | ✔️ | ✔️ | ✔️ |  |
| Møller_2020 | Subjective MVPA | Post-intervention | ✔️ | ✔️ | ✔️ | ❌ | ❌ | ✔️ | ✔️ | ✔️ | Unclear | ✔️ | ✔️ | ✔️ | ✔️ |
|  |  | 6-months |  |  |  |  |  |  |  |  |  | ✔️ | ✔️ | ✔️ |  |
| Mur-Gimeno et al. 2024 | Objective MVPA | Post-intervention | ✔️ | ✔️ | Unclear | ❌ | ❌ | ✔️ | ✔️ | ✔️ | ✔️ | ✔️ | ✔️ | ✔️ | ✔️ |
|  |  | 3-months |  |  |  |  |  |  |  |  |  | ✔️ | ✔️ | ✔️ |  |
| Mutrie et al. 2012 | Subjective TPA | Post-intervention | ✔️ | ✔️ | ✔️ | ❌ | ❌ | ✔️ | ✔️ | ✔️ | Unclear | ✔️ | ✔️ | ✔️ | ✔️ |
|  |  | 6-months |  |  |  |  |  |  |  |  |  | ✔️ | ✔️ | ✔️ |  |
|  |  | 18-months |  |  |  |  |  |  |  |  |  | ✔️ | ✔️ | ✔️ |  |
|  |  | 60-months |  |  |  |  |  |  |  |  |  | ✔️ | ✔️ | ✔️ |  |
| Nyrop et al. 2017 | Subjective Walking | Post-intervention | Unclear | Unclear | Unclear | ❌ | ❌ | ✔️ | ❌ | ✔️ | ✔️ | ❌ | ✔️ | ✔️ | ✔️ |
|  |  | 6-months |  |  |  |  |  |  |  |  |  | ❌ | ✔️ | ✔️ |  |
|  | | | | | | | | | | | | | | | |
| **Table S8.** (continued) | | | | | | | | | | | | | | | |
|  |  | **Domain** | **Selection and allocation** | | | **Administration of intervention/exposure** | | | **Assessment, detection, and measurement of outcome** | | | **Participant retention** | **Statistical Conclusion Validity** | | |
| **Study** | **Outcome** | **Result** | **1** | **2** | **3** | **4** | **5** | **6** | **7** | **8** | **9** | **10** | **11** | **12** | **13** |
| Penttinen et al. 2019 | Subjective TPA | Post-intervention | ✔️ | ✔️ | ✔️ | ❌ | ❌ | ✔️ | ❌ | ✔️ | Unclear | ✔️ | ✔️ | ✔️ | ✔️ |
|  |  | 60-months |  |  |  |  |  |  |  |  |  | ✔️ | ✔️ | ✔️ |  |
| Phillips et al. 2022 | Objective MVPA | Post-intervention | ✔️ | ✔️ | ✔️ | ❌ | ❌ | ✔️ | ❌ | ✔️ | ✔️ | ✔️ | ✔️ | ✔️ | ✔️ |
|  |  | 3-months |  |  |  |  |  |  |  |  |  | ✔️ | ✔️ | ✔️ |  |
| Pinto et al. 2008 | Subjective MVPA | Post-intervention | ✔️ | Unclear | ❌ | ❌ | ❌ | ❌ | Unclear | ✔️ | Unclear | ✔️ | ✔️ | ✔️ | ✔️ |
|  |  | 3-months |  |  |  |  |  |  |  |  |  | ✔️ | ✔️ | ✔️ |  |
| Pinto et al. 2013 | Subjective MVPA | Post-intervention | ✔️ | ✔️ | ❌ | ❌ | ❌ | Unclear | ✔️ | ✔️ | Unclear | ✔️ | ✔️ | ✔️ | ✔️ |
|  |  | 6-months |  |  |  |  |  |  |  |  |  | ✔️ | ✔️ | ✔️ |  |
| Pinto et al. 2015 | Objective MVPA | Post-intervention | Unclear | ✔️ | Unclear | ❌ | ❌ | Unclear | ✔️ | ✔️ | ✔️ | ✔️ | ✔️ | ✔️ | ✔️ |
|  |  | 3-months |  |  |  |  |  |  |  |  |  | ✔️ | ✔️ | ✔️ |  |
|  | Subjective MVPA | Post-intervention |  |  |  |  |  |  | ✔️ | ✔️ | ✔️ | ✔️ | ✔️ | ✔️ |  |
|  |  | 3-months |  |  |  |  |  |  |  |  |  | ✔️ | ✔️ | ✔️ |  |
| Pinto et al. 2022 | Objective MVPA | Post-intervention | Unclear | ✔️ | ✔️ | ❌ | ❌ | Unclear | ✔️ | ✔️ | ✔️ | ✔️ | ✔️ | ✔️ | ✔️ |
|  |  | 3-months |  |  |  |  |  |  |  |  |  | ✔️ | ✔️ | ✔️ |  |
|  | Subjective MVPA | Post-intervention |  |  |  |  |  |  | ✔️ | ✔️ | ✔️ | ✔️ | ✔️ | ✔️ |  |
|  |  | 3-months |  |  |  |  |  |  |  |  |  | ✔️ | ✔️ | ✔️ |  |
| Rogers et al. 2023 | Objective MVPA | Post-intervention | ✔️ | ✔️ | Unclear | ❌ | ❌ | Unclear | ✔️ | ✔️ | ✔️ | ✔️ | ✔️ | ✔️ | ✔️ |
|  |  | 3-months |  |  |  |  |  |  |  |  |  | ✔️ | ✔️ | ✔️ |  |
|  |  | 9-months |  |  |  |  |  |  |  |  |  | ✔️ | ✔️ | ✔️ |  |
|  | Subjective MVPA | Post-intervention |  |  |  |  |  |  | ✔️ | ✔️ | ✔️ | ✔️ | ✔️ | ✔️ |  |
|  |  | 3-months |  |  |  |  |  |  |  |  |  | ✔️ | ✔️ | ✔️ |  |
|  |  | 9-months |  |  |  |  |  |  |  |  |  | ✔️ | ✔️ | ✔️ |  |
| Schmidt et al. 2017 | Subjective TPA | Post-intervention | ✔️ | ✔️ | Unclear | ❌ | ❌ | ✔️ | Unclear | ✔️ | Unclear | ✔️ | ✔️ | ✔️ | ✔️ |
|  |  | 3-months |  |  |  |  |  |  |  |  |  | ✔️ | ✔️ | ✔️ |  |
|  |  | 12-months |  |  |  |  |  |  |  |  |  | ✔️ | ✔️ | ✔️ |  |
| Soltero_2023 | Objective TPA | Post-intervention | Unclear | Unclear | Unclear | ❌ | ❌ | ✔️ | ✔️ | ✔️ | ✔️ | Unclear | ✔️ | ✔️ | ✔️ |
|  |  | 7-months |  |  |  |  |  |  |  |  |  | Unclear | ✔️ | ✔️ |  |
|  | Subjective TPA | Post-intervention |  |  |  |  |  |  | ✔️ | ✔️ | Unclear | Unclear | ✔️ | ✔️ |  |
|  |  | 7-months |  |  |  |  |  |  |  |  |  | Unclear | ✔️ | ✔️ |  |
| Smith-Turchyn et al. 2020 | Subjective TPA | Post-intervention | ✔️ | ✔️ | ❌ | ❌ | ❌ | ✔️ | Unclear | ✔️ | ✔️ | ✔️ | ✔️ | ✔️ | ✔️ |
|  |  | 4-months |  |  |  |  |  |  |  |  |  | ✔️ | ✔️ | ✔️ |  |
| Spence et al. 2022 | Subjective TPA | Post-intervention | ✔️ | ✔️ | Unclear | ❌ | ❌ | Unclear | ❌ | ✔️ | Unclear | ✔️ | ✔️ | ✔️ | ✔️ |
|  |  | 3-months |  |  |  |  |  |  |  |  |  | ✔️ | ✔️ | ✔️ |  |
| Vallance et al. 2008 | Subjective MVPA | Post-intervention | ✔️ | ✔️ | ❌ | ❌ | ❌ | ✔️ | ❌ | ✔️ | Unclear | ✔️ | ✔️ | ✔️ | ✔️ |
|  |  | 6-months |  |  |  |  |  |  |  |  |  | ✔️ | ✔️ | ✔️ |  |
| Winters-Stone et al. 2022 | Subjective TPA | Post-intervention | ✔️ | ✔️ | Unclear | ❌ | ❌ | ✔️ | ✔️ | ✔️ | Unclear | ✔️ | ✔️ | ✔️ | ✔️ |
|  |  | 6-months |  |  |  |  |  |  |  |  |  | ✔️ | ✔️ | ✔️ |  |
| Witlox et al. 2018 | Subjective MVPA | Post-intervention | ✔️ | ✔️ | ❌ | ❌ | ❌ | ✔️ | Unclear | ✔️ | Unclear | Unclear | ✔️ | ✔️ | ✔️ |
|  |  | 4-months |  |  |  |  |  |  |  |  |  | Unclear | ✔️ | ✔️ |  |
|  |  | 44-months |  |  |  |  |  |  |  |  |  | ✔️ | ✔️ | ✔️ |  |
| *Note:* TPA=total physical activity, MVPA=moderate-to-vigorous physical activity, 1) Was true randomisation used for assignment of participants to groups?, 2) Was allocation to groups concealed?, 3) Were groups similar at baseline?, 4) Were participants blind to treatment assignment?, 5) Were those delivering the treatment blind to treatment assignment?, 6) Were groups treated identically other than the intervention of interest?, 7) Were outcome assessors blind to treatment assignment?, 8) Were outcomes measured in the same way for groups?, 9) Were outcomes measured in a reliable way?, 10) Was follow-up complete and, if not, were differences between groups in terms of their follow-up adequately described and analysed?, 11) Were participants analysed in the groups to which they were randomized?, 12) Was appropriate statistical analysis used?, 13) Was the trial design appropriate and any deviations from the standard RCT design (individual randomization, parallel groups) accounted for in the conduct and analysis of the trial? | | | | | | | | | | | | | | | |

| **Table S9.** Risk of bias assessment (quasi-experimental studies) | | | | | | | | | | | |
| --- | --- | --- | --- | --- | --- | --- | --- | --- | --- | --- | --- |
|  |  | **Domain** | **Temporal precedence** | **Selection and allocation** | **Confounding factors** | **Administration of intervention/exposure** | **Assessment, detection, and measurement of outcome** | | | **Participant retention** | **Statistical Conclusion Validity** |
| **Study** | **Outcome** | **Result** | **1** | **2** | **3** | **4** | **5** | **6** | **7** | **8** | **9** |
| Ballinger et al. 2021 | Objective Steps | Post-intervention | ✔️ | ❌ | ✔️ | ✔️ | ✔️ | ✔️ | ✔️ | ✔️ | ✔️ |
|  |  | 3-months |  |  |  |  |  |  |  | ❌ | ✔️ |
| Baumann et al. 2017 | Subjective TPA | Post-intervention | ✔️ | ✔️ | ❌ | ✔️ | ✔️ | ✔️ | Unclear | Unclear | ✔️ |
|  |  | 4-months |  |  |  |  |  |  |  | Unclear | ✔️ |
|  |  | 10-months |  |  |  |  |  |  |  | Unclear | ✔️ |
|  |  | 16-months |  |  |  |  |  |  |  | Unclear | ✔️ |
| Loo et al. 2019 | Subjective TPA | Post-intervention | ✔️ | ❌ | ✔️ | ✔️ | ✔️ | ✔️ | Unclear | ❌ | ✔️ |
|  |  | 6-months |  |  |  |  |  |  |  | ❌ | ✔️ |
|  |  | 18-months |  |  |  |  |  |  |  | ❌ | ✔️ |
| Ormel et al. 2021 | Objective MVPA | Post-intervention | ✔️ | ❌ | ✔️ | ✔️ | ✔️ | ✔️ | ✔️ | ✔️ | ✔️ |
|  |  | 3-months |  |  |  |  |  |  |  | ✔️ | ✔️ |
|  | Subjective TPA | Post-intervention |  |  |  |  | ✔️ | ✔️ | Unclear | ✔️ | ✔️ |
|  |  | 3-months |  |  |  |  |  |  |  | ✔️ | ✔️ |
| Rabin et al. 2009 | Objective TPA | Post-intervention | ✔️ | ❌ | ✔️ | ✔️ | ✔️ | ✔️ | ✔️ | ✔️ | ✔️ |
|  |  | 3-months |  |  |  |  |  |  |  | ✔️ | ✔️ |
| Schulz et al. 2022 | Subjective TPA | Post-intervention | ✔️ | ✔️ | ✔️ | ❌ | ✔️ | ✔️ | Unclear | ❌ | ✔️ |
|  |  | 12-months |  |  |  |  |  |  |  | N/A | N/A |
|  |  | 24-months |  |  |  |  |  |  |  | ✔️ | ✔️ |
| Shachar et al. 2023 | Subjective Walking | Post-intervention | ✔️ | ❌ | ✔️ | ✔️ | ✔️ | ✔️ | Unclear | ✔️ | Unclear |
|  |  | 3-months |  |  |  |  |  |  |  | N/A | N/A |
| Soleimani et al. 2016 | Subjective TPA | 3-months | ✔️ | ✔️ | Unclear | ❌ | ✔️ | ✔️ | Unclear | ✔️ | ✔️ |
| Vani et al. 2024 | Subjective MVPA | Post-intervention | ✔️ | ❌ | ✔️ | ✔️ | ✔️ | ✔️ | Unclear | ❌ | ✔️ |
|  |  | 3-months |  |  |  |  |  |  |  | ❌ | ✔️ |
| Weiner et al. 2023 | Objective MVPA | Post-intervention | ✔️ | ❌ | ✔️ | ✔️ | ✔️ | ✔️ | ✔️ | ✔️ | ✔️ |
|  |  | 3-months |  |  |  |  |  |  |  | ✔️ | ✔️ |
| Wilson et al. 2005 | Objective Steps | Post-intervention | ✔️ | ❌ | ✔️ | ✔️ | ✔️ | ✔️ | ✔️ | ❌ | Unclear |
|  |  | 3-months |  |  |  |  |  |  |  | ✔️ | Unclear |
| Note: TPA=total physical activity, MVPA=moderate-to-vigorous physical activity, 1) Is it clear in the study what is the “cause” and what is the “effect” (i.e., there is no confusion about which variable comes first)?, 2) Was there a control group?, 3) Were the participants included in any comparisons similar?, 4) Were the participants included in any comparisons receiving similar treatment/care, other than the exposure or intervention of interest?, 5) Were there multiple measurements of the outcome both pre and post the intervention/exposure?, 6) Were the outcomes of participants included in any comparisons measured in the same way?, 7) Were outcomes measured in a reliable way?, 8) Was follow-up complete and, if not, were differences between groups in terms of their follow-up adequately described and analysed?, 9) Was appropriate statistical analysis used? | | | | | | | | | | | |
